# Personal

**Published:** 1857-11

**Authors:** 


### EDITORIAL.

*Personal.*

The unceasing labors consequent on an active practice, added to college and hospital duties, renders it impossible for me to devote that time and attention to all the departments of the "North-Western Medical and Surgical Journal" which they ought to receive from the editor. To obviate this difficulty, I have secured the efficient and valuable aid of my colleague, Prof. W. H. Byford, whose name has been placed on the title-page of the present number. In addition to a more efficient superintendence of the book notices, reviews and selections, he will give frequent translations from the more valuable French and German periodicals, embodying often matter of much practical value. With his help, and the continued aid of our ordinary contributors, we expect to make the Journal much more valuable than heretofore.

All letters containing remittances, or concerning the business matters of the Journal, may be addressed as before, to N. S. Davis, Chicago, Illinois.

